# Structural insights into a 20.8-kDa tegumental-allergen-like (TAL) protein from *Clonorchis sinensis*

**DOI:** 10.1038/s41598-017-02044-0

**Published:** 2017-05-11

**Authors:** Chang Hwa Jo, Jonghyeon Son, Sulhee Kim, Takashi Oda, Jaehoon Kim, Myoung-Ro Lee, Mamoru Sato, Hyun Tae Kim, Satoru Unzai, Sam-Yong Park, Kwang Yeon Hwang

**Affiliations:** 1 Division of Biotechnology, College of Life Sciences & Biotechnology, Korea University, Seoul, 136-701 Republic of Korea; 2 Graduate School of Medical Life Sciences, Yokohama City University, Kanagawa, Japan; 3 Division of Malaria and Parasitic Diseases, Korea National Institute of Health, Osong, Republic of Korea; 4 Department of Frontier Bioscience, Faculty of Bioscience and Applied Chemistry, Hosei University, Tokyo, Japan

## Abstract

Survival of *Clonorchis sinensis*, a cause of human clonorchiasis, requires tegument proteins, which are localized to the tegumental outer surface membrane. These proteins play an important role in a host response and parasite survival. Thus, these proteins are interesting molecular targets for vaccine and drug development. Here, we have determined two crystal structures of the calmodulin like domain (amino acid [aa] positions 1–81) and dynein light chain (DLC)-like domain (aa 83–177) of a 20.8-kDa tegumental-allergen-like protein from *Clonorchis sinensis* (CsTAL3). The calmodulin like domain has two Ca^2+^-binding sites (named CB1 and CB2), but Ca^2+^ binds to only one site, CB1. The DLC-like domain has a dimeric conformation; the interface is formed mainly by hydrogen bonds between the main chain atoms. In addition, we have determined full-length structure of CsTAL3 in solution and showed the conformational change of CsTAL3 induced by Ca^2+^ ion binding using small-angle X-ray scattering analysis and molecular dynamics simulations. The Ca^2+^-bound form has a more extended conformation than the Ca^2+^-free from does. These structural and biochemical analyses will advance the understanding of the biology of this liver fluke and may contribute to our understanding of the molecular mechanism of calcium-responsive and tegumental-allergen-like proteins.

## Introduction

*Clonorchis sinensis* is a parasite from the class of human liver flukes and causes human clonorchiasis. It is heavily endemic in Southern China (including Hong Kong and Taiwan), Korea, Japan, and other Southern Asian countries^1, 2^. It is currently estimated that more than 200 million people are at risk of infection and ~20 million are infected globally^3, 4^. Humans are mainly infected via consumption of undercooked (including dried, salted, smoked, or pickled) or raw infected fish^2, 5–8^. The symptoms of human clonorchiasis include indigestion, fullness of the abdomen, loss of appetite, epigastric distress unrelated to meals, diarrhea, edema, hepatomegaly, and toxemia from liver impairment. The most serious consequence of clonorchiasis is that it has been implicated in cholangiocarcinoma in mammals including humans^2–4, 9–14^. The control of clonorchiasis relies on treatment with a single drug, praziquantel^15^. Despite its efficacy, safety, and low cost, this drug induces several adverse reactions, such as abdominal pain, diarrhea, dizziness, sleepiness, headache, and there is a possibility of development of resistance in parasites. The most important limitation of praziquantel is that it does not prevent reinfection^6, 16–18^.

The tegumental outer surface of blood-dwelling flatworms is a unique double-bilayer membrane structure that is crucially important for survival of the parasite in the face of humoral immune responses^19^. Tegumental proteins, localized to the tegumental outer surface membrane, play a role in parasite–host interactions such as nutrient transport, environmental signal transduction, and evasion of host’s immune system^20–22^. The Ca^2+^-binding protein family of tegumental proteins was predicted to have unique composition and structure that consists of a calmodulin like domain and dynein light chain (DLC)-like domain^23–25^. This unique structure does not exist in mammalian proteins, and the function is unknown^26, 27^. This tegumental protein family affects immune responses and exerts its influence via a number of EF-hand motifs. Therefore, this protein family has been named tegument-allergen-like (TAL)^28–31^. Tegumental protein of 20.8 kDa from *Clonorchis sinensis* elicits IgA immune responses in the host and does not cause an IgG response^32, 33^. This characteristic is similar to that of SmTAL3 (20.8-kDa tegumental protein from *Schistosoma mansoni*, sequence identity 38%, positives 60%)^30, 31^. For this reason, we named the 20.8-kDa tegumental protein from *Clonorchis sinensis* as CsTAL3.

Signalling by calcium ions is important in living system such as parasites. The most common related in calcium signalling motif is the EF-hand motif which is the best characterized in calmodulin^34^. Several antagonist of calmodulin, chlorpromazine (CPZ), Trifluoperazine (TPZ) and Phenothiazine (PTZ), were used in the treatment psychotic disorders^35–37^. Moreover, the tegumental proteins, such as SmTAL1,2,3 and CsTALs, is localized in host-interactive layer that has accessibility of selecting target molecules for vaccines and drugs^38^. Thus, the tegumental proteins are one of the most interesting molecular targets for development of vaccines and drugs^32, 39^.

In this work, we determined 2.6 Å crystal structure of the DLC-like domain (amino acid [aa] positions 83–177) and 1.3 Å crystal structure of the calmodulin like domain (aa positions 1–81) of CsTAL3. Furthermore, we present the full-length structure of CsTAL3 in solution state and its conformational change upon Ca^2+^ binding using small-angle X-ray scattering (SAXS) analysis. Our results should improve the understanding of the biology of liver flukes and may contribute to the development of new vaccines and drugs against clonorchiasis.

## Results and Discussion

### Overall structure of DLC-like domain of CsTAL3

At first, we tried crystalizing full-length CsTAL3 (aa 1–184), but the crystal structure contained only the DLC-like domain (aa 83–177). The interesting thing is that similar results were reported for SmTAL2 and FhCaBP2^27, 40^. Both proteins belong to the TAL protein family of the class of fluke proteins that consist of a calmodulin like domain (or N-terminal domain) and a DLC-like domain (or C-terminal domain) as in CsTAL3. We also confirmed that CsTAL3 is completely cleaved into two domains in constant buffer condition (20 mM Tris/HCl, pH 7.5, 100 mM NaCl, 1 mM DTT) after ~20 days at 20 °C with various Ca^2+^ ion concentration (Supplementary Fig. 1). The cleavage mechanism of the flexible linker of these proteins shows instability of proteins and may be a general property *in vivo* ^27^. As a result, the selenium-methionine-derivatized crystal of the DLC-like domain of CsTAL3 (aa 83–177) diffracted to 2.8 Å resolution and was found to belong to space group *P2*_*1*_ *2*_*1*_ *2* with six protomers in the asymmetric unit. The initial phase determination and model building were accomplished by the SAD method with anomalous signals of 18 selenomethionines. The native crystal of the DLC-like domain of CsTAL3 diffracted to 2.6 Å resolution and belongs to space group *C222*_*1*_ with three molecules per asymmetric unit. For determining the structure of the N-terminal domain (calmodulin like domain) of CsTAL3, later, we also attempted to crystallize only this domain (aa 1–81), and next, the resulting crystal diffracted to 1.3 Å resolution (see Table 1).

**Table 1:** Data collection and refinement statistics for CsTAL3.

	DLC-like domain (SeMet)	DLC-like domain	Calmodulin like domain (SeMet)	Calmodulin like domain
**Data collection**				
Space group	P2_1_2_1_2	C222_1_	P4_1_	P4_1_
Cell dimensions				
*a*, *b*, *c* (Å)	61.0, 88.4, 152.9	88.4, 154.1, 61.6	48.1, 48.1, 42.6	47.8, 47.8, 42.5
α, β, γ (°)	90, 90, 90	90, 90, 90	90, 90, 90	90, 90, 90
Resolution (Å)	50–2.80 (2.80–2.85)	50–2.6 (2.68–2.59)	50–2.04 (2.08–2.04)	50–1.30 (1.35–1.30)
Completeness (%)	99.3 (97.6)	94.5 (77.6)	99.7 (99.7)	99.5 (99.0)
Redundancy	8.1 (5.2)	8.2 (5.6)	13.0 (10.9)	10.4 (6.8)
I/σ (I)	24.0 (2.1)	23.2 (3.4)	87.4 (25.1)	27.1 (2.5)
R_merge_ (%)	8.6 (32.5)	8.9 (64.3)	5.5 (12.2)	3.4 (37.8)
**Refinement statistics**				
Resolution (Å)		48.13-2.60		47.83-1.30
R_work_/R_free_ (%)		22.4/26.8		18.0/21.6
B-factor (Averaged)				
Protein		96.3		28.6
Ca^2+^				17.6
Water		71.3		44.8
R.m.s. deviations				
Bond lengths (Å)		0.01		0.01
Bond angles (°)		1.58		1.29
Ramachandran plot (%)				
favored		98		100
allowed		2		0
PDB ID		5X2D		5X2E

Highest resolution shell is shown in parentheses.

**Figure 1:**
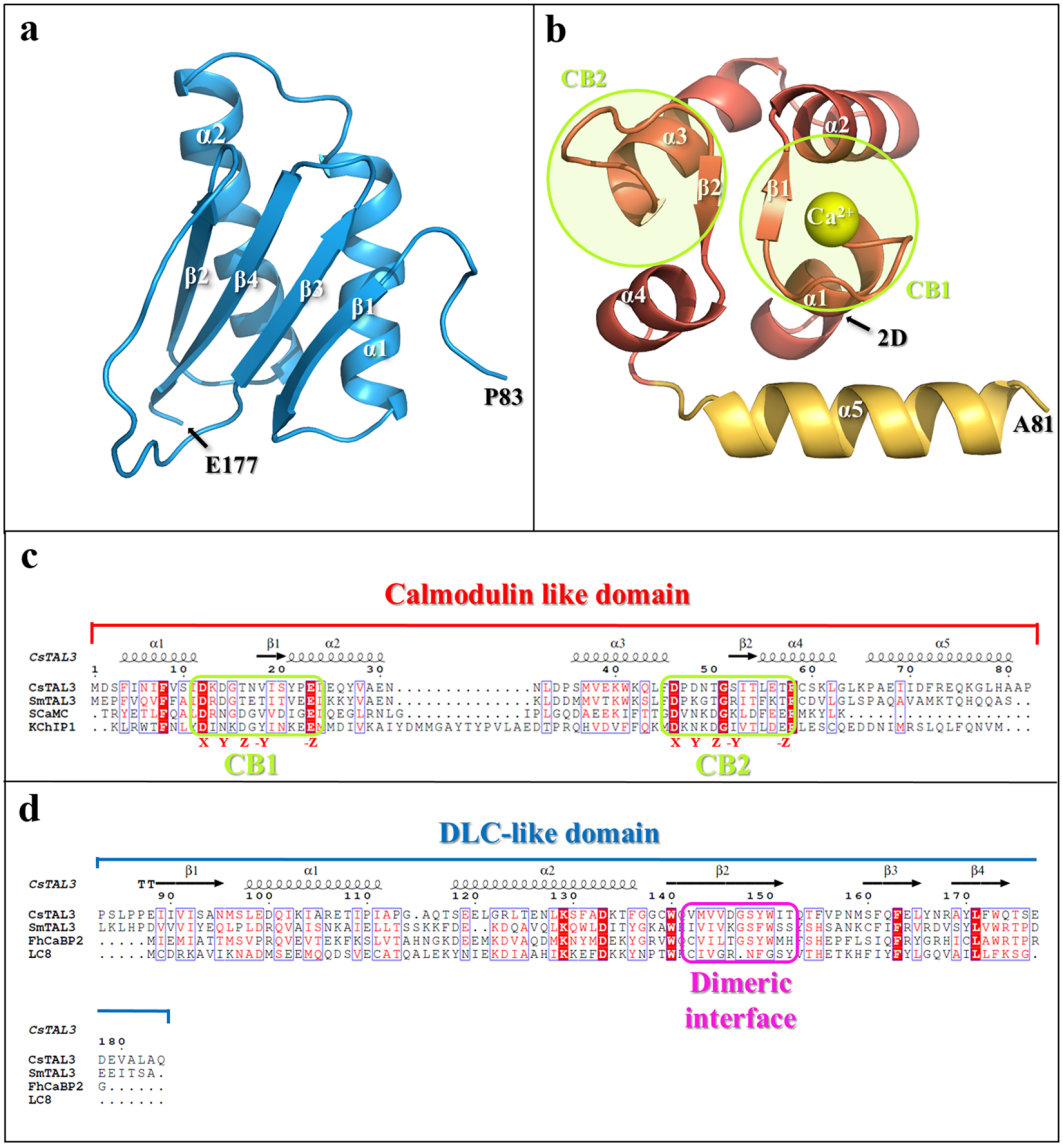
Overall crystal structure of CsTAL3 and sequence alignment. **(a)** Monomeric structure of the DLC-like domain (aa 83–177). **(b)** Crystal structure of the calmodulin like domain (aa 2–81). *Green* circles represent Ca^2+^-binding domains 1 and 2 (CB1 and CB2). **(c)** Sequence alignment of the calmodulin like domain of CsTAL3 from *Clonorchis sinensis* (accession code: Q2PMV7), SmTAL3 from *Schistosoma mansoni* (P91804), SCaMC1 from *Homo sapiens* (Q6NUK1), and KChIP1 from *Homo sapiens* (Q9NZI2). CB1 and CB2 residues are boxed in *green*, and Ca^2+^-binding positions are labeled under each residue. **(d)** Sequence alignment of the DLC-like domain of CsTAL3, SmTAL3, and FhCaBP2 from *Fasciola hepatica* (A0A0B5GUS3), and LC8 from *Homo sapiens* (P63167). Dimeric-interface residues are boxed in *magenta*.

**Figure 2:**
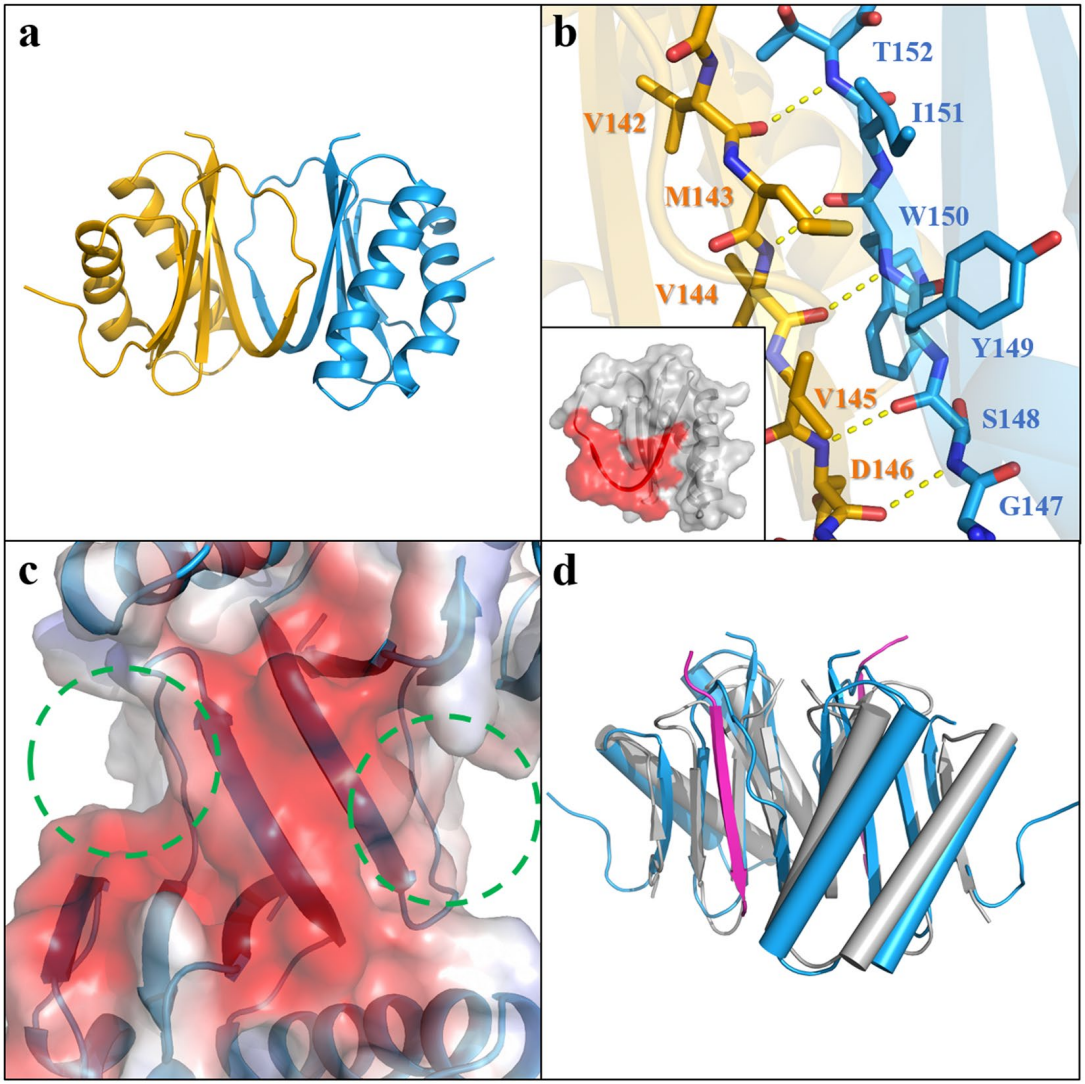
Dimeric structure of the DLC-like domain and of its dimeric interface. **(a)** Dimeric structure of the DLC-like domain. One monomer is colored in *sky-blue*, and the other in *gold*. **(b)** Close-up view of the dimeric interface. *Yellow* dashed lines represent hydrogen bonds of the main chains. In the box, the dimeric-interface area is represented by *red*. The dimeric interface is on average ~17.4% in the solvent-accessible area. **(c)** The electrostatic surface of the DLC-like domain dimer. The *green* dashed circle represents the hydrophobic groove. **(d)** Superimposition of the DLC-like domain dimer (*sky-blue*) and LC8 dimer (*gray*) complexed with a peptide of NEK9 (*magenta*) (PDB code: 3ZKE).

The monomeric structure of the DLC-like domain consists of four anti-parallel β-strands that are packed with each other and an extended loop protruding from β-sheets; the other face of the β-sheets is packed with two α-helices (Fig. 1a). Despite low sequence identity, the DLC-like domain of CsTAL3 shows structural similarities with the DLC-like domain of FhCaBP2 and 8-kDa human dynein light chain (LC8) with root mean square (r.m.s.) deviation 0.79 and 2.65 Å when 69 Cα and 49 Cα atoms are aligned in Pymol^41^, respectively (Supplementary Fig. 2). The dimeric interface information calculated by PISA^42^ is that the dimeric interface area is on average ~1044 Å^2^ (17.4%) at the total solvent-accessible area of 6033 Å^2^, and the solvation free energy gain upon formation of the interaction is on average −15.2 kcal/mol (Fig. 2b). The DLC-like domain of CsTAL3 has a dimeric conformation similar to that of some DLC domains, which are LC8 (3ZKF, r.m.s. deviation 2.0 Å when 82 Cα atoms are aligned, DALI server^43^ Z-score is 12.3), dynein light chain from *Saccharomyces cerevisiae* (Dyn2; 4DS1, r.m.s. deviation 2.3 Å when 84 Cα atoms are aligned, Z-score is 12.0), and human dynein light chain 2 (DYNLL2; 2XQQ, r.m.s. deviation 2.1 Å when 82 Cα atoms are aligned, Z-score is 11.9). The five residues (G147 to T152) of each protomer in the extended loop interact with five residues (V′142 to D′146) of the neighboring protomer in the β_2_-strand via a pair of hydrogen bonds. Side chains of these strands also contribute to the hydrophobic interaction (Fig. 2b). Nonetheless, despite the conservation of the structural characteristics, the amino acid sequences of the dimeric interface are not conserved relative to the other DLC families (Fig. 1c). These findings suggest that dimeric interactions of the DLC-like domain are determined only by hydrogen bonds of the main chain^27^. Protein partners of LC8 interact with the extended β-sheet of the LC8 homodimer with backbone hydrogen bonds and side chain interactions. In the structure of the LC8 complex with peptide of Nek9 (PDB: 3ZKE, 3ZKF), the peptide interacts with the hydrophobic groove of the LC8 dimer; this groove is composed of β1, β3, β4, and α2′ ^43–45^. The DLC-like domain of CsTAL3 also contains a hydrophobic groove (Fig. 2c). The superimposition of LC8 with the peptide and DLC-like domain shows a similar conformation (Fig. 2d). This result suggests that CsTAL3 may interact with its binding partner proteins in a similar manner.

### Overall structure of Calmodulin like domain of CsTAL3

Due to cleavage of the full-length protein, we had grown a crystal of the calmodulin like domain (aa 1–81) of CsTAL3. The crystal diffracted to 1.3 Å resolution and belongs to space group *P4*_*1*_ with one molecule per asymmetric unit. The initial phase determination and model building were carried out by the SAD method with an anomalous signal of one selenomethionine. The molecule shows structural similarities with the calmodulin like domain of a family of soluble Ca^2+^ sensor Kv-channel-interacting proteins (KChIPs) and of the short Ca^2+^-binding mitochondrial carrier (SCaMC) with r.m.s. deviation 1.8 Å and 2.8 Å, when 60 Cα and 62 Cα atoms are aligned, and DALI server Z-scores 8.2 and 8.1^46^, respectively (Supplementary Fig. 3).

**Figure 3:**
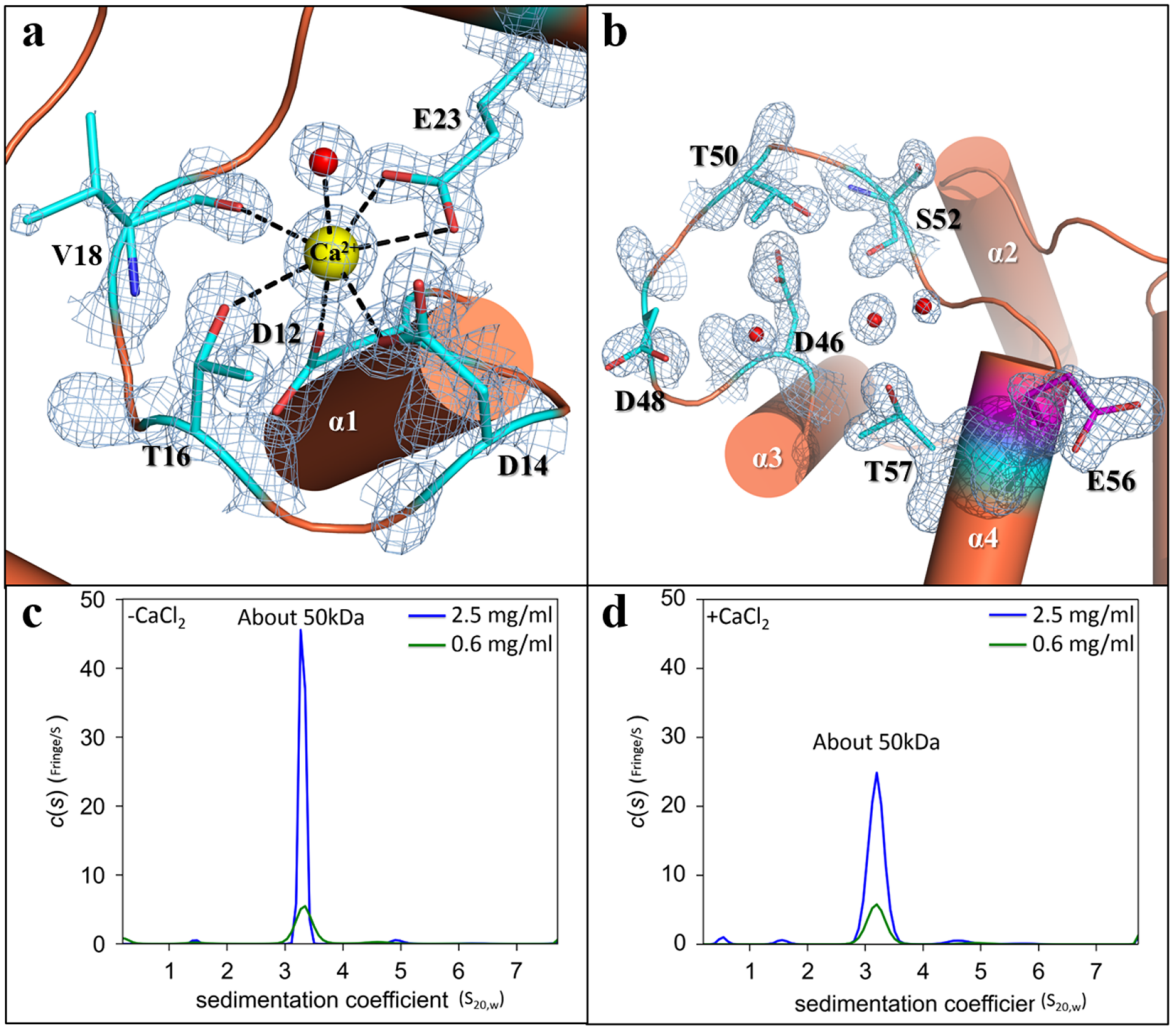
Structure of CB1 and CB2, and analytical ultracentrifugation analysis of CsTAL3 induced by a Ca^2+^ ion. **(a**,**b)** Close-up view of the structure and the electro density map of CB1 and CB2. The *yellow* molecule represents a Ca^2+^ ion and *red* molecules represent water molecules. **(c**,**d)** Results of analytical ultracentrifugation analysis of CsTAL3 induced by CaCl_2_. The X direction means a sedimentation coefficient at 20 °C in pure water (**S**_**20,w**_), and the Y direction means continuous distribution, **c(s)**.

The structure of the calmodulin like domain is composed of five α-helices. The α1 to α4 helices are classical EF-hand motifs where two helix-loop-helix structures and two short antiparallel β-sheets (β1 and β2) are connecting the Ca^2+^-binding loops (Fig. 1b). Ca^2+^-binding motif 1 (CB1, residues 12–23) is a Ca^2+^-binding loop that contains 12 partially conserved residues starting with N-terminal aspartate and ending with C-terminal glutamate as in the EF-hand motif of other calmodulin like proteins (Fig. 1b and c). One Ca^2+^ ion binds to CB1 via D12, D14, T16, V18, E23, and a water molecule (positions X, Y, Z, −Y, −Z, and −X, respectively) in a geometrical pattern of a pentagonal bipyramid. Other residues bind a Ca^2+^ ion via their side chain carboxyl groups, but V18 (–Y position) binds to a Ca^2+^ ion via its main-chain carbonyl oxygen atom (Fig. 3a). Recently, structure of SmTAL3 was predicted that does not bind a Ca^2+^ ion according to various biochemical experiments such as by limited proteolysis, native gel electrophoresis, differential scanning fluorimetry, and dot blots with radioactive calcium ions^25, 47^. However, the CB1 of SmTAL3 sequences is highly conserved relative to CB1 of other calmodulin like proteins as SCaMC, KChIP1 and CsTAL3 (Fig. 1c). Although the –Y position sequence different, this is not a problem for the Ca^2+^-binding property because a Ca^2+^ ion is bound only by the main-chain carbonyl oxygen atom of the –Y position residue. Considering this, we propose the possibility of Ca^2+^-binding property in the CB1 of SmTAL3.

Residues of Ca^2+^-binding motif 2 (CB2, aa 46–57) are predicted to be D46, D48, T50, S52, and T57 from the sequence alignment with the EF-hand motif of KChIP1 and SCaMC (Fig. 1c). Moreover, CB2 structure appears to be similar to that of CB1, but a Ca^2+^ ion is absent in our structure. A significant difference is that the –Z position of CB1. This position is glutamate in the other EF-hand motif, but the –Z position of CB2 is threonine (T57). Although threonine is also a polar amino acid, it is not accessible to the Ca^2+^-binding region (Fig. 3b).

To confirm that the dimeric interaction of CsTAL3 is affected by the conformational change of the backbone folding with Ca^2+^ binding in CB1, we performed AUC analyses. The c(s) distribution of CsTAL3 shows the presence of a single species with a sedimentation coefficient (s_20,w_) of 3.3 ± 0.1 S (without CaCl_2_) and 3.2 ± 0.1 S (+5 mM CaCl_2_). The molecular weight of the single species corresponds to ~50 kDa with and without a Ca^2+^ ion (Fig. 3c and d). These results suggest that the dimeric form of CsTAL3 is stable and its dimerization state is not affected by the Ca^2+^ binding in CB1. The c(s) distribution peak shape of without CaCl_2_ is much sharper than that of with CaCl_2_. These results proposed that CsTAL3 structure changes upon Ca^2+^ ion binding and the c(s) peak shape reflects the CsTAL3 structural changes.

### Determination of the full-length structure of CsTAL3 in solution

**Figure 4:**
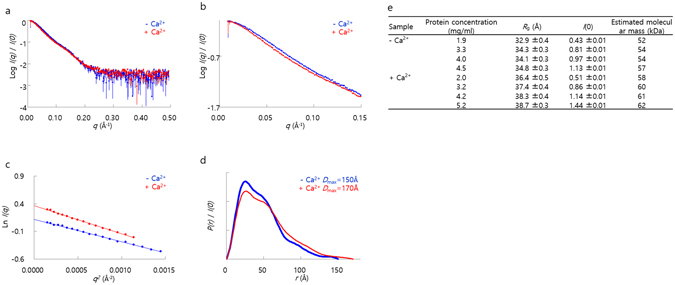
Small-angle X-ray scattering (SAXS) analysis of CsTAL3 induced by a Ca^2+^ ion. **(a**,**b)** The experimental *I*(*q*) at the highest protein concentration and the close-up view of *I*(*q*). The experimental *I*(*q*) is shown as dots with error bars. **(c)** The Guinier plot at the highest protein concentration. **(d)** The distance distribution function *P*(*r*) at the highest protein concentration. **(e)** A summary of *R*_g_, *I*(0), and molecular mass estimated from the Guinier plot.

To analyze the conformational change of *CsTAL3* induced by Ca^2+^ binding, we performed SAXS measurements. Scattering intensity I(*q*) was obtained in the protein concentration range 1.9 to 5.2 mg/mL. The Guinier plot indicated that the protein solution used in the SAXS analysis did not contain any aggregates (Fig. 4c). Estimated molecular mass of CsTAL3 was approximately range ~50 to ~60 kDa, indicating that CsTAL3 exists as a homodimer in solution (Fig. 4e). I(*q*) was slightly but unambiguously changed in a *q*-range < 0.15 Å^−1^ (Fig. 4b). The radius of gyration (R_g_) of the Ca^2+^-bound form was larger than that of the Ca^2+^-free form (Fig. 4e). Distance distribution function *P*(*r*) of CsTAL3 showed a broader distribution with a shoulder ~55 Å, which is a characteristic of noncompact flexible proteins composed of two domains (Fig. 4d). There are a slightly broader shoulder of *P*(*r*) and larger R_g_ in the Ca^2+^-bound form than those of in the Ca^2+^-free form. These results indicate that the distance between the calmodulin like domain and DLC-like domain in the Ca^2+^-bound form seems to be slightly larger than that in the Ca^2+^-free form.

**Figure 5:**
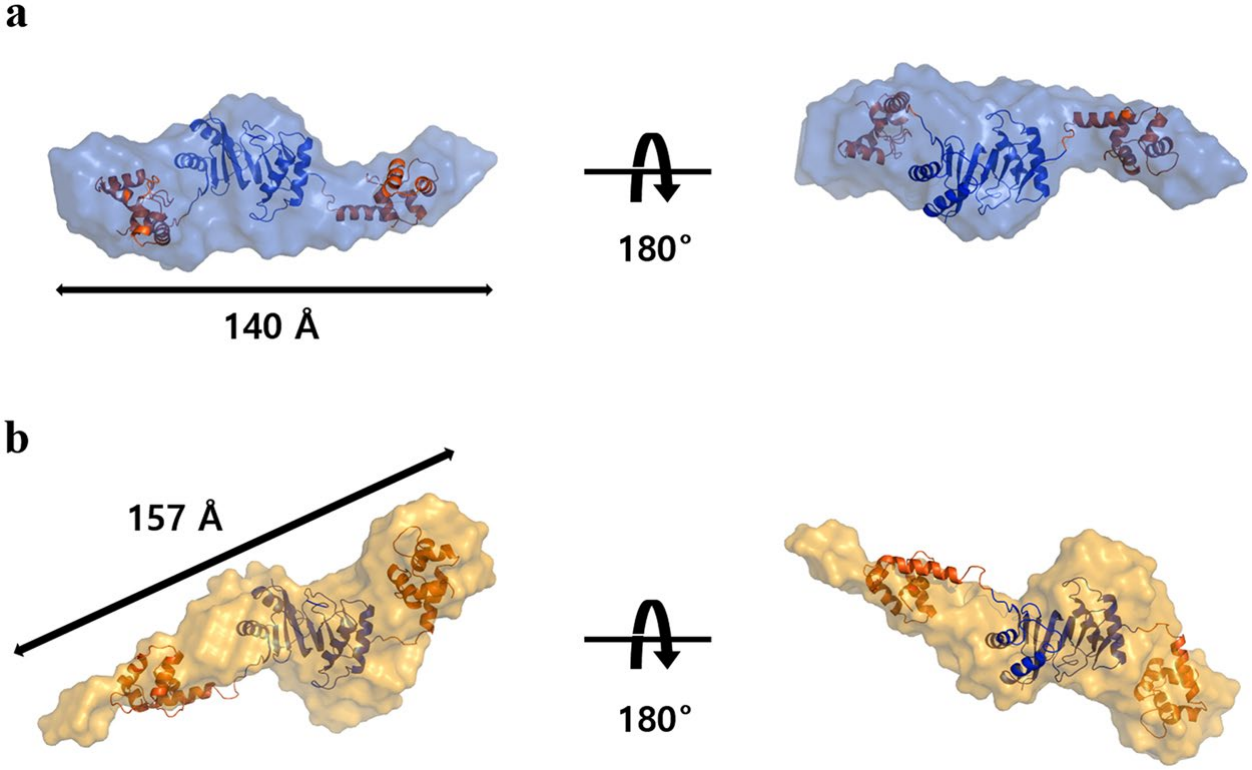
Best-fit *ab initio* envelopes for the full-length structure of CsTAL3 in solution state. Envelopes generated by DAMMIF are displayed in translucent **(a)** *sky-blue* (Ca^2+^-free form) and **(b)** *gold* (Ca^2+^-bound form).

BILBOMD is rigid body modeling with molecular dynamics simulations; it generates the minimal ensemble for the best agreement with the experimental scattering data^48^. Although full-length crystal structure was not obtained here, we determined the full-length structure of CsTAL3 in solution and analyzed the conformational change of CsTAL3 induced by Ca^2+^ binding by means of BILBOMD. Residues 1–80 of the calmodulin like domain and residues 88–177 of the DLC-like domain were defined as fixed, while residues 81–87 (the unstructured portion between the two domains) were defined as flexible in our BILBOMD analysis. Despite setting large R_g_ ranges (20–50 Å) for molecular dynamics simulations, each resulted in calculation of R_g_ only between 30 and 34 Å. Thus, we estimated that flexibility is limited between the DLC-like domain and calmodulin like domain. The best-fit models of the Ca^2+^-bound form and Ca^2+^-free form have R_g_ ≈ 32 Å. The theoretical scattering profile for each model is in good agreement with the experimental scattering data (χ^2^ = 1.8 and 2.6). According to the results of the bead modeling, linear length of the Ca^2+^-bound form is ~157 Å, which is longer than ~140 Å of the Ca^2+^-free form. In the molecular dynamics simulation models, the distance between the two domains corresponds to the more extended conformation in the Ca^2+^-bound form than in the Ca^2+^-free form (Fig. 5). This result indicates that the calmodulin like domain and flexible linker undergo a conformational change upon Ca^2+^ binding. These modeling results are consistent with AUC results. Thus, we propose that the structure of CsTAL3 changes to the extended conformation upon Ca^2+^ binding; this conformational change may play a role in parasitic worms. This structural information should improve the understanding of the unique Ca^2+^-binding tegumental proteins and may facilitate the development of new drugs or vaccines. Thus, more research is needed on the exact function and mechanism of action of this protein *in vivo* to understand the physiological processes of parasitic worms.

## Materials and Methods

### Protein expression and purification

The gene of full-length (aa 1–184) CsTAL3 (UniProt ID: Q2PMV7) was cloned into pRSET-b (Merck Millipore, GE). The recombinant vector was transfected into *Escherichia coli* BL21(DE3) and B834(DE3) (Merck Millipore, GE). The cells were grown at 37 °C in the Luria-Bertani medium and in the M9 minimal medium containing 60 μg/ml L-selenomethionine with 100 μg/ml ampicillin up to optical density (at 600 nm) of 0.6. The protein expression was induced by the addition of 1 mM isopropyl-D-thiogalactopyranoside at 18 °C with incubation for 18 h. The cell pellet was resuspended in ice-cold lysis buffer consisting of 20 mM Tris-HCl (pH 7.5), 100 mM NaCl, 5 mM CaCl_2_, and 2 mM β-mercaptoethanol. After sonication and centrifugation for 1 h at 17364 × *g* and 4 °C, the supernatant was loaded onto a HiTrap Chelating HP column (GE Healthcare, USA). The recombinant protein was eluted using a linear gradient of 1 M imidazole added to lysis buffer. The fractions was incubated at 4 °C for a hour in the buffer included 5 mM EGTA to remove any bounded calcium ions. Further purification was conducted on a HiLoad 16/600 Superdex 200 prep-grade column (GE Healthcare, USA) with a buffer consisting of 20 mM Tris-HCl (pH 7.5), 100 mM NaCl, ±5 mM CaCl_2_, and 1 mM dithiothreitol. The fractions containing the purified protein were pooled and concentrated to 20 mg/mL using an Amicon Ultra Centrifugal Filter (Merck Millipore, GE), which was stored at −80 °C prior to crystallization trials.

The calmodulin like domain (aa 1–81) of CsTAL3 was cloned into pET-21a (Merck Millipore, GE). It was also transfected, expressed, and purified using the same protocols and buffering conditions as described above for the full-length CsTAL3 protein.

### Crystallization and data collection

Initial protein crystal screen of full-length CsTAL3 was carried out by the sitting-drop vapor diffusion method at 22 °C. After a month, microcrystals were obtained in 200 mM MgSO_4_ and 20% polyethylene glycol 3350. These crystallization conditions were optimized by the hanging-drop vapor diffusion method at 22 °C. The suitable crystals of native and selenium-methionine-derivatized version were grown in 170 mM MgSO_4_ and 21% polyethylene glycol 3350. The crystals were frozen in liquid nitrogen with 15% (w/v) ethylene glycol as a cryoprotectant. The selenium-methionine-derivatized crystals were grown under the same conditions and in the same cryoprotectant as the native crystals were. X-ray diffraction data were collected using a wavelength 1.1000 Å on beamline BL-1A at the Photon Factory (Tsukuba, Japan).

Crystals of the calmodulin like domain of CsTAL3 were prepared by the same protocol as we used for full-length CsTAL3. The initial crystals were obtained in 100 mM sodium acetate and 3 M NaCl. The suitable crystals of the native and selenium-methionine-derivatized version for X-ray diffraction analysis were grown in 90 mM sodium acetate and 3.25 M NaCl. The crystals were frozen in liquid nitrogen with 4.8 M NaCl as a cryoprotectant. X-ray diffraction data on the native and selenium-methionine-derivatized crystals were collected using a wavelength 0.9800 Å on beamline BL-17A at the Photon Factory (Tsukuba, Japan) and on beamline 5C-SBII at the Pohang Light Source (Pohang, Korea), respectively. The raw data were indexed, integrated, and scaled using the HKL2000 software suite^49^. Crystallographic statistics of data collection are provided in Table 1.

### Structure determination

The initial phases were obtained from the selenium-methionine single-wavelength anomalous dispersion (SAD) dataset using AutoSol in software package PHENIX^50^. Further structure was determined by molecular replacement based on the initial model of selenium-methionine data using PHASER in PHENIX^51^. The model building and refinement were performed using the *Coot* ^52^ and PHENIX^51^. The structure was validated with MolProbity^53^. The statistics of structure refinement are provided in Table 1. The coordinates and structure factor of the DLC-like domain and calmodulin like domain were deposited in the Protein Data Bank with the accession codes 5X2D and 5X2E, respectively.

### Structural analysis

The structure-based sequence alignment was generated using Clustal Omega^54^ and ESPript^55^. Root mean square (r.m.s.) deviation and Z-score of structure alignment were calculated using the DALI server^46^. The dimeric interface area and free energy of dissociation were calculated in PISA^42^. All images of the crystal structure were generated using PyMol^41^.

### Analytical ultracentrifugation (AUC) experiment

The experiments were conducted at 20 °C using an Optima XL-I analytical ultracentrifuge (Beckman Coulter, USA) with an An-50 Ti rotor. For sedimentation velocity experiments, cells with a standard Epon two-channel centerpiece and sapphire windows were used. The sample (400 μL) and reference buffer (420 μL) were loaded into the cells. The rotor temperature was equilibrated at 20 °C in the vacuum chamber for 1–2 h prior to the startup. The sedimentation velocity experiment was conducted at protein concentrations of 2.5 and 0.6 mg/mL. Changes in the concentration gradient were monitored with a Rayleigh interference optical system at 10-min intervals during sedimentation at 50 × 10^3^ rpm. Partial specific volume of the protein, solvent density, and solvent viscosity were calculated from standard tables using the SEDNTERP software^56^. The resulting scans were analyzed using the continuous distribution c(s) analysis module in the SEDFIT software^57^. Sedimentation coefficient increments of 100 were used in the appropriate range for each sample. The frictional coefficient was allowed to float during fitting. The weighted average sedimentation coefficient was obtained by integrating the range of sedimentation coefficients in which peaks were present. The values of the sedimentation coefficient were corrected to 20 °C in pure water (s_20,w_). The c(s) distribution was converted into c(M), a molar mass distribution.

### Small Angle X-ray Scattering (SAXS)

SAXS measurements were performed at 20 °C on a BioSAXS-1000 system (Rigaku, Japan) mounted on a MicroMax007HF X-ray generator (Rigaku, Japan). The PILATUS 100k detector, at a sample-to-detector distance of 482.8 mm, was used to measure scattering intensities. Sample solutions in 20 mM Tris-HCl pH 7.5 with 100 mM NaCl were used for SAXS measurements. The samples containing 5 mM CaCl_2_ were used for analysis of the Ca^2+^-bound form. Circular averaging of the scattering intensities was carried out by means of the SAXSLab software (Rigaku, Japan) to obtain one-dimensional scattering data I(q) as a function of q (q = 4πsinθ/λ, where 2θ is the scattering angle, and the X-ray wavelength λ = 1.5418 Å). To check the interparticle interference, I(q) data were collected at different protein concentrations (1.9, 3.3, 4.0, and 4.5 mg/mL for the Ca^2+^-free form; 2.0, 3.2, 4.2, and 5.2 mg/mL for the Ca^2+^-bound form). To estimate molecular mass of CsTAL3, SAXS measurements of standard proteins (5.8 mg/mL glucose isomerase [172 kDa], 1.6 mg/mL BSA [66 kDa], 5.0 mg/mL ovalbumin [43 kDa], and 2.7 mg/mL hen egg lysozyme [14 kDa]) were carried out under the same conditions. Exposure time was 2 h for CsTAL3, BSA, and lysozyme and 0.5 h for glucose isomerase and ovalbumin. All SAXS data were analyzed with the software applications embedded in the ATSAS package^58^. The radius of gyration R_g_ and forward scattering intensity I(0) were estimated from the Guinier plot of I(q) in a smaller-angle region of qR_g_ < 1.3^59^. The distance distribution function P(r) was calculated by means of the GNOM software^60^, where the experimental I(q) data were used in a q-range from 0.011 to 0.303 Å^−1^. The maximum particle dimension D_max_ was estimated from the P(r) function as the distance r for which P(r) = 0^60^. Bead-modeling software DAMMIF^61^ was used to generate an *ab initio* model. Ten individual runs of DAMMIF were conducted and averaged with DAMAVER^62^. BILBOMD was used for rigid body modeling by molecular dynamics simulations and minimal ensemble model generation^48^. Initial models of full-length CsTAL3 for BILBOMD analysis were generated with *Coot* ^52^. The collected SAXS data and data statistics are provided in Fig. 4.

## Electronic supplementary material

Supplementary Information
