# Supplementary material for: Structural insights into a 20.8-kDa tegumental-allergen-like (TAL) protein from Clonorchis sinensis
Source: Sci Rep. 2017 May 11;7:1764. doi: 10.1038/s41598-017-02044-0 (PMC5431922; doi:10.1038/s41598-017-02044-0)
Supplement: Supplementary file 1 — Supplementary Information [file 41598_2017_2044_MOESM1_ESM.pdf]

Supplementary Data

Structural insights into a 20.8-kDa tegumental-allergen-like (TAL) protein from *Clonorchis sinensis*

Chang Hwa Jo[a], Jonghyeon Son[a], Sulhee Kim[a], Takashi Oda[b], Jaehoon Kim[a], Myoung-Ro Lee[a,c], Mamoru Sato[b], Hyun Tae Kim[a], Satoru Unzai[d], Sam Yong Park[b], and Kwang Yeon Hwang[a]*

[a]Division of Biotechnology, College of Life Sciences & Biotechnology, Korea University, Seoul 136-701, Republic of Korea

[b]Graduate School of Medical Life Sciences, Yokohama City University, Kanagawa, Japan

[c]Division of Malaria and Parasitic Diseases, Korea National Institute of Health, Osong, Republic of Korea

[d]Department of Frontier Bioscience, Faculty of Bioscience and Applied Chemistry, Hosei University, Tokyo, Japan

*Corresponding author. Division of Biotechnology, College of Life Sciences & Biotechnology, Korea University, Seoul 136-701, Republic of Korea. Tel.: +82 2-3290-3009, Fax: +82 2-923-3225; E-mail: chahong@korea.ac.kr (K.Y. Hwang).

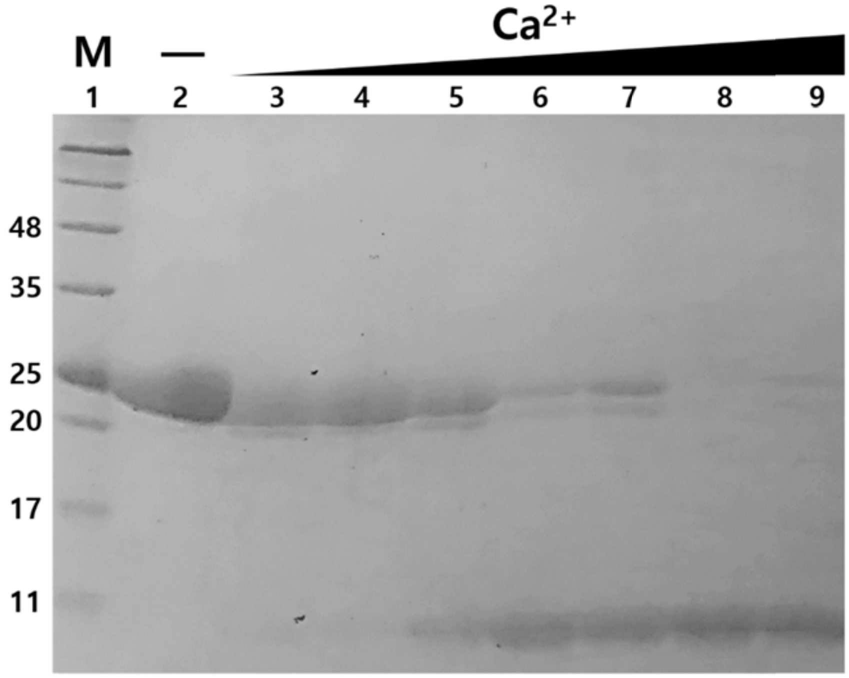

Supplementary Figure 1. SDS-PAGE of CsTAL3 with various calcium concentration. All sample was incubated in constant buffer condition (20 mM Tris/HCl, pH 7.5, 100 mM NaCl, 1 mM DTT) at 20°C during about 20 days with various calcium concentration. Lane 1, molecular-weight marker (labelled in kDa); lane 2, negative control protein of CsTAL3; lane 3 to 8, molar ratio gradient of $CaCl_2$ concentration in buffer condition, concentration of CsTAL3 : calcium concentration (1 : 0.5, 1 : 1, 1 : 2, 1 : 5, 1 : 10, 1 : 15, and 1 : 20).

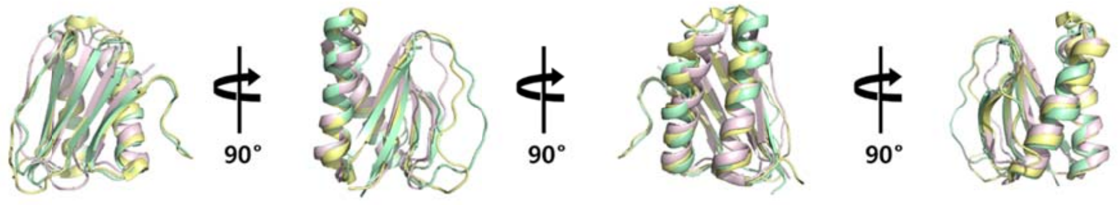

Supplementary Figure 2. Superimposition of the DLC-like domains from Clonorchis sinensis CsTAL3 (*green*), *Fasciola hepatica* FhCaBP2 (*yellow*, RMSD was 0.79 Å in 69 Cα, PDB code : 5FX0) and *Homo sapiens* LC8 (*pink*, RMSD was 2.65Å in 49 Cα, 3ZKE).

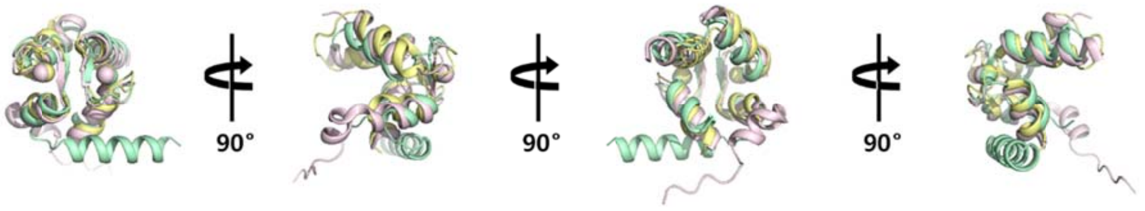

Supplementary Figure 3. Superimposition of the calmodulin like domains from *Clonorchis sinensis* CsTAL3 (*green*), *Homo sapiens* SCaMC (*yellow*, RMSD was 2.8 Å in 62 Cα, 4N5X), *Homo sapiens* KChIPs (*pink*, RMSD was 1.8 Å in 60 Cα, 2I2R).

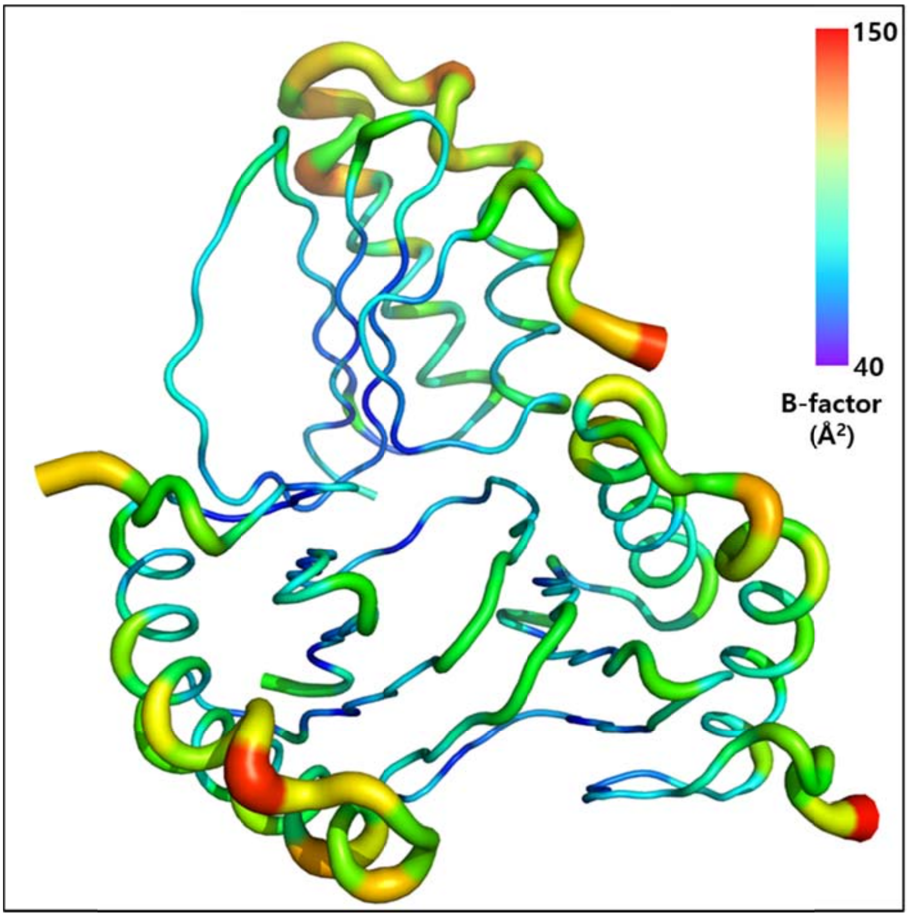

Supplementary Figure 4. Crystallographic trimeric structure of CsTAL3 color-coded by atomic B-factors. The B-factor value is color-coded on a blue-to-red spectrum.
